# Physically distant but socially close? Changes in non-physical intergenerational contacts at the onset of the COVID-19 pandemic among older people in France, Italy and Spain

**DOI:** 10.1007/s10433-021-00621-x

**Published:** 2021-04-26

**Authors:** Bruno Arpino, Marta Pasqualini, Valeria Bordone

**Affiliations:** 1 Department of Statistics, Computer Science, Applications,, University of Florence, Viale Morgagni, 59, 50134 Firenze, Italy; 2 Observatoire Sociologique du Changement (OSC), Sciences Po, Paris, France; 3 Department of Sociology, University of Vienna, Rooseveltplatz 2, 1090 Vienna, Austria

**Keywords:** COVID-19, Digital communication, Intergenerational contacts, Intergenerational relationships, Social contacts

## Abstract

Physical distancing is intended to mitigate the spread of the SARS-CoV-2 virus. However, the impact of a decrease in face-to-face contacts on non-physical social contacts of older people remains unclear. In particular, we focus on intergenerational contacts that are especially relevant for older people’s mental health. Our analyses rely on an online quota sampling survey conducted in France, Italy and Spain during April 14–24, 2020. We considered the subsample of individuals aged 50 + (*N* = 4207). We calculated post-stratification weights based on official statistics and used logistic regressions to analyze how changes in intergenerational contacts differed by socioeconomic factors and to what extent non-physical contacts (via phone, social media, etc.) have compensated the reduction in face-to-face contacts. Finally, the change in digital devices’ use has been explored as a consequence of both decreased physical intergenerational contacts (PIC) and increased non-physical intergenerational contacts (NPIC). We found that about 50% of older people have increased their NPIC during the first lockdown. Younger individuals, those with medium level of education, and those economically better off displayed higher probabilities of increased NPIC as compared to their counterparts. NPIC increased especially for individuals whose face-to-face contacts decreased, particularly so if this happened with respect to contacts with children. A large share of older people has increased their use of video calls and instant messages, while only some increased the use of social media. These findings are relevant to understand how intergenerational contacts changed during the pandemic and may be central to better plan future outbreak responses.

**Supplementary Information:**

The online version contains supplementary material available at 10.1007/s10433-021-00621-x.

## Introduction

Since the beginning of the COVID-19 pandemic, limitations to physical (i.e., face-to-face) contacts have been imposed among other nonpharmaceutical interventions and guidelines devoted at fighting the spread of the SARS-CoV-2 virus. These, together with other direct and indirect consequences of the pandemic, likely had a negative impact on older people’s mental health (e.g., Brooks et al. 2020; Sheffler et al. 2020). In such a context, non-physical intergenerational contacts (e.g., via mobile phones) may have played a particularly important role in limiting the negative effects of reduced physical contacts (Arpino et al. 2020a).

This paper focuses on intergenerational contacts for three reasons. First, intergenerational (family) contacts constitute a large part of older people’s overall relationships (Dykstra 2018). Second, they represent a central source of emotional and physical support for older people (Antonucci et al. 2007; Cooney and Dykstra 2013). Third, intergenerational contacts have been considered as a crucial factor in contributing to the spread and lethality of COVID-19 across different areas (Bayer and Kuhn 2020).

In particular, we aim at exploring the heterogeneity in changes in non-physical intergenerational contacts (NPIC) according to older people’s characteristics and at examining whether—and to what extent—older people’s NPIC have increased, possibly to replace physical intergenerational contacts (PIC) with non-coresident individuals. Finally, we study whether increased frequency of NPIC stimulated an increased use of means of communications that are usually less common among older people (e.g., video calls, instant text messaging, social media).

Our research is based on a timely online survey carried out in France, Italy and Spain in April 2020, during the first lockdown. At the time of implementing the survey, these countries represented a particularly interesting setting because they were the first outside Asia to be strongly hit by the pandemic (Ceylan 2020) and to lock down completely to reduce the contagion (Hale et al. 2020). As part of the strict measures implemented, physical contacts have consistently been reduced in all three countries, especially among older people (Del Fava et al. 2020). While these three countries are particularly relevant for studying how and for whom NPIC have increased during the first lockdown, differences between countries are not the focus of this paper. Hence, the multivariate analyses will be carried out pooling data and controlling for country dummy variables.

## Background

### The COVID-19 pandemic and changes in social contacts

One of the most evident consequence of the interventions and guidelines implemented to address the COVID-19 pandemic has been the reduction of physical contacts (Del Fava et al. 2020). As the COVID-19 is particularly deadly for older people (Guan et al. 2020), especially those with pre-existing health conditions (Clark et al. 2020), policy and media discourses have encouraged older people to limit their movements and physical contacts (Ayalon 2020), putting them at high risk of isolation. In particular, intergenerational (physical) contacts have been suspected to be potentially risky for the transmission of the virus to older people. However, Arpino et al. (2020b), while explaining the nonclearcut evidence on the macrolevel association between physical intergenerational relationships and the diffusion of the virus, argued that intergenerational relationships are a multidimensional system that does not exclusively include physical contacts (Bengtson and Roberts 1991; Tomassini et al. 2004). Thus, NPIC may have become more common during the lockdown as an instrument to enforce associational solidarity and as a way to deal with stress and depressive feelings (Arpino et al. 2020a).

### Changes in non-physical intergenerational contacts (NPIC) during the first wave of the COVID-19 pandemic: Research questions

From the discussion in the previous section, it emerges that during the COVID-19 pandemic older people have been invited or forced to reduce their physical contacts (whether intergenerational or not) and empirical evidence has shown that they in fact did so. Our first research question is therefore: (RQ1) *To what extent have NPIC, instead, increased? And, are changes in such contacts different in different groups?*

Previous studies have largely shown that strong heterogeneities in intergenerational contacts existed before the onset of the COVID-19 pandemic within countries. Such differences may be found across gender, age and socioeconomic status. For example, women tend to have more frequent social contacts, in general, than men (Barawnoska-Rataj and Abramowska‑Kmon 2019) and people’s social contacts tend to decline with age (Barawnoska-Rataj and Abramowska‑Kmon 2019) although intergenerational contacts acquire an even higher importance in later life (Antonucci et al. 2014). A wide array of studies have also shown that higher education tends to be associated with lower likelihood of weekly face-to-face contacts between adult children and their older parents (Grundy and Shelton 2001).

To what extent the known heterogeneities in intergenerational contacts will also manifest in changes in NPIC during the pandemic is difficult to predict. Thus, we take an exploratory approach, empirically accounting for most of the factors that are usually considered among the correlates of intergenerational contacts. Additionally, heterogeneous changes in NPIC during the pandemic are also expected to be linked to persistent technological divides (by age, education, etc.), which do not only refer to Internet access, but also to technological attitudes, skills and types of usage (van Deursen and van Dijk 2015). Our empirical analyses are based on an online survey, thus targeting a selected group of older people with access to the Internet. Still within this group both the frequency of use of digital communications and their type may vary. Previous studies have shown that among individuals aged 50 and over, younger age, being men, higher education, and more economic resources are associated with higher levels of digital literacy and with more regular use of the Internet (Halmdienst et al. 2019; van Deursen and van Dijk 2015), although these differences have somewhat reduced over time (Huxhold et al. 2020).

Our second research question is: (RQ2) *Do reduced PIC associate with an increase in NPIC among older people?* Baldassar et al. (2016) argue that physical and non-physical contacts do not differ in terms of their power in generating the sense of a shared experience of everyday family lives. According to existing studies on transnational families, regular non-physical contacts may reinforce the feeling of belonging to a family network, thus decreasing feelings of isolation (Nedelcu and Wyss 2016; Nedelcu 2017). Older people may have increased NPIC during the first COVID-19-related lockdown to (partially) compensate for the reduction in physical contacts, which has subtracted a relevant source of emotional and practical support.

Our third question asks: (RQ3) *With what ties have non-physical contacts increased?* More specifically, we distinguish between contacts with (grand)children and (grand)parents for those who have at least one family member alive of the corresponding type of tie. The intergenerational relationships literature has documented continued and intensive contacts with children (Grundy and Read 2012), which also serve as a bridge for contacts with grandchildren (Hank and Buber 2009). Therefore, we may expect especially non-physical contacts with children to have grown during the first lockdown to compensate for reduced physical contacts.

Finally, our fourth question is: (RQ4) *What type of digital communication form has mostly increased?* In particular, we investigate changes in the use of video calls, instant messaging applications, and communication via social media and whether these changes are associated with changes in NPIC.

## Methods

### Study population

We implemented an online survey targeting the population aged 18 and older in France, Italy and Spain. In this study, we focus on the subsample of individuals aged 50 + . The survey has been carried out through the online market survey platform called Lucid that offers the opportunity to purchase samples for survey research, showing to produce high-quality and rigorous data (Coppock and McClellan 2019). More specifically, Lucid recruits respondents following a three-step process (Callaghan et al. 2019) starting with the collection of lists of potential survey respondents invited to take part in the panel. Then, on individuals who agreed to participate, Lucid collects a general set of variables (e.g., gender, age, education, income) and, finally, based on this information, individuals are invited to participate to a particular survey using quota sampling. This sampling implies splitting the population into a number of subsets according to the characteristics of interest and in a way that the proportion of each subset in the sample is the same as in the population. This procedure ensures that the final sample is nearly identically distributed as the country benchmark given by statistics provided by national statistical offices.

As recommended in online survey research, we kept the questionnaire short to minimize non-responses and false responses (Revilla and Ochoa 2017). More specifically, we set up a 10-min questionnaire focussed on changes in PIC and NPIC during the lockdown.

Because online surveys can only involve individuals who are online, and who agreed to become part of a panel as well as to take part in the specific study (Duffy et al. 2005), a coverage bias consisting in a misrepresentation of the target population might emerge. However, representativeness was guaranteed by defining the sample quotas in terms of a significant set of variables (gender, age, region of residence, education). Additionally, we have used post-stratification weights to minimize deviations from the benchmark population statistics. Specifically, we used the STATA package “ipfweight” (Bergmann 2011), to generate weights through an iterative procedure that performed stepwise adjustments of the weights until it has achieved, within countries, the margins of three population distributions: region of residence, age and gender, age and education. Clearly, the sampling and post-stratification adjustments can only guarantee representativeness of the sample with respect to these important socio-demographic variables.

The target sample was of 3000 individuals per country (9000 in total). When restricting the sample to individuals aged 50 + , the total sample size was reduced to 4207 observations. Data were collected between April 14 and 24, 2020. The first nationwide lockdown restrictions were implemented around mid-March in all three countries (Italy: March 10, Spain: March 14; France: March 17, 2020).

We excluded from the analytic sample individuals who have increased PIC (*N* = 439) and those who had decreased NPIC (*N* = 435) during the lockdown (note that 23 individuals satisfied both exclusion criteria). In fact, both groups are rather small to allow for analyses with sufficient power. We also tried keeping them in the dataset and results did not substantially change. Table S.1 in *Supplementary Materials* shows the distribution of the original sample according to changes in NPIC and PIC.

More information on the survey is available at a dedicated website (https://sites.google.com/unifi.it/intergen-covid; see Arpino et al. 2020c for descriptive results on the full sample and the questionnaire).

### Dependent variables

Individuals were asked about changes in their NPIC (e.g., video calls, instant text messages, social media) during the first COVID-19 lockdown with non-coresident individuals distinguishing between parents, grandparents, children and grandchildren. This is used as a categorical variable taking value 1 if respondents increased the frequency of NPIC with at least one non-coresident family member of different generation since the entry into force of the first nationwide restrictions due to the COVID-19 and 0 if they maintained them unchanged.

In addition, we consider a similar categorical variable for contacts with specific family members: children, grandchildren, and parents and/or grandparents (because the number of respondents reporting contacts with grandparents is limited (57), for simplicity in the following we label the latter category as “parents” only).

Then, we consider if respondents increased (= 1) or not (= 0) NPIC via specific means of communication: video calls, instant messaging applications, and social media. Although for these variables we do not have specific information on with whom respondents have increased their use, subsequent analyses have been conducted on subsamples based on whether respondents have or not specific family ties: children, grandchildren, and parents in order to shed further light on these changes.

### Independent variables

Similar to NPIC, individuals were asked about changes in their PIC during the first COVID-19 lockdown up to the time of interview. Thus, we include a categorical variable taking value 1 if respondents decreased the frequency of PIC with at least one non-coresident family member of another generation during the COVID-19 lockdown and 0 otherwise. In addition, we account for the reduction in PIC specifically with children, grandchildren, and parents.

### Control variables

Controls include socio-demographic variables such as respondents’ gender, age, country of residence (France, Italy or Spain), subjective economic situation (living comfortably on present income; coping on present income; finding it difficult on present income; finding it very difficult on present income) and the availability of kin (parents, children, grandchildren). We also controlled for the level of education (three levels based on the International Standard Classification of Education—low = below secondary education, medium = up to high school and high = university education or above) and for whether respondents were, or not, employed in the pre COVID-19 period. In addition, we included two health-related variables with regard to the period antecedent to the COVID-19 pandemic: respondents’ self-perceived health (0 = very good or good; 1 = fair, poor or very poor) and any reported chronic diseases (such as heart diseases, hypertension, stroke or cancer). An additional variable accounted for the severity level with which the region where the respondent lives was hit by the pandemic according to the tertiles of the distribution of case fatality rates (CFRs) of COVID-19 at the regional level (NUTS-22).

In a robustness check (Table S.2 in *Supplementary Material*), we also added additional control variables accounting for events experienced during the COVID-19 pandemic, obtaining similar results. There, a set of dummy variables accounted for whether respondents have experienced each of the following events since the beginning of the lockdown up to the interview time: reduction in physical activity; worsened relation with partner; worsened relation with other people; suffered income loss; lost job; difficulties with organizing work or study from home; death of a relative or friend due to coronavirus; a relative or friend was infected; had more time to spend with family; made new friends; re-established a relationship with a relative or friend; and my life was not affected a big deal.

### Statistical analyses

We first produced descriptive analyses on the main variables. Then, we used logistic regressions to examine associations between socio-demographic and health-related individuals’ characteristics and the probability of increased NPIC with at least one non-coresident family member of a different generation during the lockdown considered (Model 1; RQ1). Then, we added to the model a measure capturing any decrease in PIC (Model 2; RQ2). Subsequently (RQ3), we differentiated intergenerational contacts into specific relationships as those with children for respondents who are parents (Model 2a; *N* = 2397), grandchildren for those who are grandparents (Model 2b; *N* = 1105), and contacts with parents for those who have at least one of them alive (Model 2c; *N* = 1109). Then, additional analyses (RQ4) have been conducted to explore the association between changes in both PIC and NPIC and the use of video calls (Model 3), instant messages (Model 4) and social media (Model 5). Finally, estimates have been replicated for subsamples to differentiate intergenerational contacts into specific relationships as those with children (Model 3a/4a/5a; *N* = 2397), grandchildren (Model 3b/4b/5b; *N* = 1105), and parents (Model 3c/4c/5c; *N* = 1109).

Average marginal effects (AMEs) have been computed to complement estimated coefficients reported in regression tables, as well as predicted probabilities that are reported graphically for the main independent variables in the *Supplementary Material* (Figures S.1-S.5). In a logistic regression, the AME of a numerical independent variable measures the effect of increasing that variable by one unit on the predicted probability. In other words, the AME in this case is equivalent to the difference in the predicted probabilities calculated at two consecutive values of the independent variable. Similarly, for a categorical variable the AME is the difference between the predicted probabilities for individuals belonging to a certain group minus the predicted probability of individuals belonging to the group representing the reference category. Post-stratification weights were used in all the analyses.

## Results

Weighted descriptive statistics for the main variables of interest are reported by country in Table 1. These statistics are calculated on the working sample, where the abovementioned exclusion restrictions have been applied. Table S.3 in *Supplementary Material* includes descriptive statistics for the other variables. Concerning changes in PIC, about 50% of respondents reduced the frequency of face-to-face contacts. PIC have been reduced especially with children (33%), while the proportion of individuals aged 50 + who reduced physical contacts with parents during the lockdown is around 18%.

**Table 1:** Descriptive Statistics on the explanatory variables (%)

Explanatory variables	Categories	Total	France	Italy	Spain
*Non-physical contacts*					
Intergenerational (all)	Increased	49.88	49.68	46.82	53.83
Intergenerational (children)	Increased	35.45	38.31	31.44	37.31
Intergenerational (grandchildren)	Increased	21.20	22.16	19.07	22.80
Intergenerational (parents and/or grandparents)	Increased	18.55	19.76	16.27	20.07
*Physical contacts*					
Intergenerational (all)	Decreased	48.53	47.26	48.36	50.09
Intergenerational (children)	Decreased	32.95	35.64	29.86	33.89
Intergenerational (grandchildren)	Decreased	25.62	26.55	26.36	23.75
Intergenerational (parents and/or grandparents)	Decreased	18.31	18.15	17.99	18.89
*Use of media/digital devices*					
Use of video calls	Increased	45.26	35.57	48.32	51.79
Use of instant messages	Increased	53.83	38.09	56.94	66.70
Use of social media	Increased	27.12	22.32	27.87	31.27

*N* = 3,333. Post-stratification weights are used. Source: Intergen-COVID online survey. Data were collected between April 14 and 24, 2020

Findings show that 50% of respondents aged 50 + increased their NPIC during the lockdown and this has mainly been with children (35%) and grandchildren (21%).

Finally, with respect to changes in the use of digital forms of communications, a consistent share of respondents aged 50 + increased the use of video calls (45%) and instant messages (54%) during the lockdown, while the increased use of social media has been reported by less than a third of respondents (27%).

Table 2 displays the results from logistic regressions assessing associations between the probability of increased NPIC during the first COVID-19 lockdown and individual’s socio-demographic and health characteristics (Model 1; RQ1). Findings from Model 1 show a nonlinear relationship between age and the probability of increased NPIC during the lockdown (in fact the coefficient of the quadratic term is statistically significant at the 5% level). However, as shown in Figure S.1 in *Supplementary Material*, within the range of observed ages the relationship is monotonically negative. In addition, respondents with a medium educational level are about 8 percentage points more likely to report increased frequency of NPIC compared to those with a higher educational level (*p* < 0.05). Finding it difficult to live on present income is associated with a lower probability of increased frequency of NPIC of about 7 percentage points (*p* < 0.001) compared to coping with present income. In addition, respondents in a partnership are about 5 percentage points more likely to have increased NPIC compared to those without a partner (*p* < 0.001; Figure S.2a). The probability of increased NPIC during the considered lockdown was about 21 percentage points higher among respondents with at least one parent alive (*p* < 0.001; Figure S.2b). Finally, compared to parents without grandchildren, (grand)childless respondents are about 28 percentage points less likely to have increased virtual contacts during the lockdown. However, this gap further increases (by 14 pp) for grandparents (Figure S.2c).

**Table 2:** Association between individuals’ characteristics and non-physical intergenerational contacts during the lockdown (Model 1)

Variables	Model 1	
	Beta (SE)	AMEs (SE)
Age	− 0.180* (0.099)	− 0.037* (0.020)
Age^2	0.001** (0.000)	^a^
Gender: Female (ref. Male)	0.076 (0.100)	0.015 (0.020)
Educational level: Medium (ref. High)	0.391** (0.177)	0.081** (0.036)
Educational level: Low (ref. High)	0.303 (0.212)	062 (0.043)
Country: Italy (ref. Spain)	− 0.287** (0.133)	− 0.059** (0.027)
Country: France (ref. Spain)	− 0.254* (0.144)	− 0.052** (0.029)
Income: Living comfortably on present income (ref. Coping on present income)	− 0.096 (0.140)	− 0.019 (0.029)
Income: Finding it difficult on present income (ref. Coping on present income)	− 0.338*** (0.124)	− 0.070*** (0.025)
Income: Finding it very difficult on present income (ref. Coping on present income)	− 0.139 (0.209)	− 0.028 (0.043)
Self-rated health: Poor (ref. Good)	− 0.112 (0.113)	− 0.023 (0.023)
Chronic diseases: Yes (ref. No)	0.142 (0.114)	0.029 (0.023)
Employment status: Yes (ref. No)	0.072 (0.115)	0.014 (0.023)
Childless & Grandchildless (ref. Children only)	− 1.364*** (0.138)	− 0.289*** (0.026)
Children & Grandchildren (ref. Children only)	0.631*** (0.126)	0.142*** (0.027)
Parents alive	1.021*** (0.101)	0.210*** (0.019)
Partner alive	0.287**(0.118)	0.059**(0.024)

*N* = 3,333. Post-stratification weights are used. Standard Error (SE) in brackets; AMEs: Average Marginal Effects. (a) The AME for age is the average effect over the age distribution; the AME of age squared is meaningless per se. Source: Online survey implemented by the authors. Data were collected between April 14 and 24, 2020

Results from Model 2 (RQ2; Table 3) show that the decrease of PIC during the first COVID-19-related lockdown among individuals aged 50 + was positively associated with an increased frequency of NPIC. Predicted probabilities drawn from Model 2 show that respondents who have decreased PIC were about 43 percentage points more likely to have increased virtual contacts (*p* < 0.001; Figure S.3 in *Supplementary Material*).

**Table 3:** Association between changes in physical intergenerational contacts and non-physical intergenerational contacts during the lockdown (Model 2, Model 2a, Model 2b and Model 2c)

Variables			Subgroup: Parents		Subgroup: Grandparents		Subgroup: Individuals with at least one parent and/or grandparent alive	
	Model 2		Model 2a		Model 2b		Model 2c	
	beta (SE)	AMEs (SE)	beta (SE)	AMEs (SE)	beta (SE)	AMEs (SE)	beta (SE)	AMEs (SE)
Decreased physical intergenerational contacts (all)	2.072*** (0.111)	0.435*** (0.021)						
Decreased physical intergenerational contacts (children)			1.985*** (0.133)	0.433*** (0.026)				
Decreased physical intergenerational contacts (grandchildren)					1.556*** (0.182)	0.351*** (0.037)		
Decreased physical intergenerational contacts (parents and/or grandparents)							1.908*** (0.151)	0.420*** (0.030)

Model 2, *N* = 3,333; Model 2a, *N* = 2,397; Model 2b, *N* = 1,105; Model 2c, *N* = 1,109. Post-stratification weights are used. Control variables not shown.. Robust standard errors (SE) in parentheses; AMEs: Average Marginal Effects ***p < 0.01, **p < 0.05, *p < 0.1

Online survey implemented by the authors. Data were collected between April 14 and 24, 2020

Subsequent models in Table 3 address RQ3 by showing findings of logistic regressions implemented on specific subsamples to distinctly look at contacts with children (Model 2a), grandchildren (Model 2b), and parents (Model 2c). Overall, the association between decreased PIC and increased NPIC is strong and statistically significant, but it shows a meaningful heterogeneity based on with whom the frequency of contacts has changed. Specifically, we found that—among parents—respondents who decreased the frequency of physical contacts with children were about 43 percentage points more likely to have increased virtual contacts with them during the lockdown. Similar findings were found for contacts with parents (AMEs = 0.420; *p* < 0.001), while the predicted probability of increased non-physical contacts with grandchildren was 35 percentage points higher for those who decreased physical contacts with them if respondents were grandparents (AMEs = 0.351; *p* < 0.001; see also Figure S.4a–4c in *Supplementary Material*).

Finally, to address RQ4 we have estimated the association between changes in NPIC and PIC and the increased use of digital communications during the lockdown (Table 4). Results from adjusted logistic regressions show that respondents who have increased NPIC were about 21 percentage points more likely to have increased video calls (Model 3), 15 percentage points more likely to have increased the use of instant messages (Model 4) and about 13 percentage points more likely to have increased the use of social media (Model 5). By distinctly looking at the frequency of contacts with different family ties according to whether respondents have or not these ties, we noticed that the increased frequency of non-physical contacts with grandchildren was particularly relevant: they were 22 percentage points more likely to have increased use of video calls (Model 3b) and 11 percentage points more likely to have increased use of instant messages and social media (Models 4b and 5b).

**Table 4:** Association Between Changes in Intergenerational physical and non-physical Contacts and Increased Use of Media/Digital Devices During the Lockdown (Model 3, Model 4 and Model 5; Model 3a, b and c; Model 4a, b and c; Model 5a, b and c)

Variables	Subgroups		Model 3 (video calls)		Model 4 (instant messaging applications)		Model 5 (social media)	
			Beta (SE)	AMEs (SE)	Beta (SE)	AMEs (SE)	Beta (SE)	AMEs (SE)
Increased non-physical intergenerational contacts (all)	Whole sample		1.099*** (0.109)	0.215*** (0.019)	0.729*** (0.112)	0.152*** (0.022)	0.718*** (0.120)	0.133*** (0.022)
Decreased physical intergenerational contacts (all)			0.450*** (0.109)	0.091*** (0.022)	0.466*** (0.113)	0.099*** (0.024)	0.0183 (0.127)	0.003 (0.023)
Increased non-physical intergenerational contacts (children)	Subgroup: Parents	a	1.056*** (0.132)	0.215*** (0.025)	0.781*** (0.133)	0.162*** (0.027)	0.650*** (0.143)	0.125*** (0.028)
Decreased physical intergenerational contacts (children)			0.294** (0.136)	0.060** (0.027)	0.455*** (0.137)	0.094*** v	− 0.093 (0.144)	− 0.017 (0.027)
Increased non-physical intergenerational contacts (grandchildren)	Subgroup: Grandparents	b	1.153*** (0.189)	0.221*** (0.032)	0.567*** (0.176)	0.114*** (0.034)	0.653***(0.167)	0.113*** (0.029)
Decreased physical intergenerational contacts (grandchildren)			0.446** (0.181)	0.085** (0.034)	0.591*** (0.176)	0.119*** (0.034)	0.194 (0.175)	0.033 (0.030)
Increased non-physical intergenerational contacts (parents and/or grandparents)	Subgroup: Individuals with at least one parent and/or grandparent alive	c	0.804*** (0.154)	0.166*** (0.030)	0.618*** (0.156)	0.131*** (0.032)	0.402** (0.164)	0.082** (0.033)
Decreased physical intergenerational contacts (parents and/or grandparents)			0.195 (0.154)	0.040 (0.031)	0.262 (0.156)	0.055 (0.033)	0.201 (0.158)	0.041 (0.032)

Model 3, Model 4, Model 5, *N* = 3,333; Model 3a, Model 4a, Model 5a, *N* = 2,397; Model 3b, Model 4b, Model 5b, *N* = 1,105; Model 3c, Model 4c. Model 5c, *N* = 1,109. Robust standard Error (SE) in parentheses; AMEs: Average Marginal Effects

Post-stratification weights are used. Control variables not shown. ***p < 0.01, **p < 0.05, *p < 0.1. Source: Online survey implemented by the authors. Data were collected between April 14 and 24, 2020

Respondents who decreased PIC during the lockdown were about 10 percentage points more likely to have increased video calls (AMEs = 0.091; *p* < 0.001; Fig. S.5a in *Supplementary Material*) and instant messages (AMEs = 0.099; *p* < 0.001; Fig. S.5b). However, a nonstatistically significant association was found between having decreased PIC and the use of social media (AMEs = 0.003; *p *> 0.1; Fig. S.5c). Moreover, our findings show that the predicted probability of increased video calls and instant messages was particularly high among grandparents who have decreased physical contacts with grandchildren (AMEs = 0.085; *p* < 0.05 and AMEs = 0.119; *p* < 0.001, respectively).

## Conclusion

Nonpharmaceutical interventions adopted at the beginning of the COVID-19 pandemic to contrast the diffusion of the SARS-CoV-2 virus had a strong impact on social contacts. We examined how NPIC have changed during the first national lockdown due to the pandemic in three countries: France, Italy and Spain. Our analyses are based on the subsample of individuals aged 50 + of an online survey carried out during the lockdown in April 2020.

We addressed four research questions. First, we asked to what extent NPIC have increased during the first lockdown and whether changes in these contacts have been heterogeneous across subgroups. Related to this broad research question, we in particular examined whether NPIC increased mostly among older people who have reduced their PIC. Consistent with other research (e.g., Del Fava et al. 2020), our data show that PIC have decreased during the lockdown for about 50% of older people in all three countries analyzed. NPIC have instead increased, but differently for different groups of older people (RQ1). Individuals in the younger ages considered, with medium level of education, and with a better (subjective) economic situation displayed higher probabilities of increased NPIC as compared to their counterparts. NPIC increased especially for those who decreased the frequency of face-to-face contacts (RQ2).

Our third research question (RQ3) examined with what ties NPIC have increased the most. We found a particularly substantial increase in NPIC between parents and children among parents who decreased the amount of physical contacts with their children during the lockdown.

Finally, we explored changes in NPIC by type of digital communication form (RQ4). A large share of older people have increased their use of video calls and instant messages. This sort of “catching-up” effect is similar to what has been observed in studies on digital divides showing narrowing gaps by age and education over time (Huxhold et al. 2020). However, only a smaller part of respondents declared to have increased their use of social media during the considered lockdown.

Older people that increased NPIC were more likely to increase the use of digital forms of communications as compared to those who did not increase NPIC. Although we do not have information on the persons contacted through such digital tools, the existing evidence suggests that the goal of increasing intergenerational contacts at a distance during the pandemic has stimulated a higher use of digital devices, consistently with what has been found in pre-COVID studies (Huxhold et al. 2020). This effect was particularly strong for those who increased non-physical contacts with grandchildren and, in terms of tools, for video calls. This may be due to the fact that video calls allow to manifest affect in a way that resembles physical contacts (Peng et al. 2018; Quadrello 2005). Existing studies on the role of digital devices as a tool to shorten geographical distance between (grand)parents and (grand)children have shown that, although non-physical, virtual contacts may reinforce family ties, emotional support and solidarity between generations by increasing older people’s agency with regard to family decisions and actions (Nedelcu and Wyss 2016; Nedelcu 2017). A lower increase in the use of social media as compared to other technologies may also be related to a resistance against this tool by groups of older people (Vulpe and Crăciun 2020).

Limitations of our study include the fact that, being based on an online survey, our results refer to Internet users and may not generalize to the whole population of older people. It may be that older people that were not using the Internet before the pandemic started to use digital forms of communications during the COVID-19 pandemic, so their NPIC may have increased even more than for their counterparts already connected. Alternatively, it may be that Internet nonusers found it more difficult to increase their social contacts at a distance. Furthermore, we could not specifically identify changes in the use of telephone calls. Future studies may investigate these relevant aspects.

Another limitation derives from the need of keeping the questionnaire short. We were not able to account for some aspects that may be explored in future studies such as the frequency of contacts before and during the lockdown. Also, the data used here have been collected during the early phase of the COVID-19 pandemic and future studies should examine whether our findings also apply to other phases of the pandemic. Moreover, in this study we do not consider country differences, so future studies should examine country heterogeneity in relation to normative and institutional differences related to the role of the family. Finally, although intergenerational family contacts constitute a large share of older people’s contacts with non-coresident individuals, and because of the growing importance of nonfamily intergenerational ties (Bordone and Arpino 2021), other studies may account also for changes in social contacts more generally, or focus specifically on individuals living alone.

Despite some limitations, our study demonstrates that the COVID-19 pandemic produced relevant indirect impacts on intergenerational contacts. On the one hand, physical distancing had a negative impact on face-to-face intergenerational contacts. On the other hand, social distancing implied by less face-to-face contacts has been compensated by more non-physical social contacts between (grand)children and (grand)parents. Our study also highlights important heterogeneities within the older population in respect to intergenerational contacts at a distance. These results have several important implications for researchers and policy makers. While models of intergenerational solidarity stress the importance of considering its complex and multidimensional nature, physical co-presence is considered as a necessary condition to realize some forms of intergenerational solidarity, such as provision of personal care, but not other, such as the provision of emotional support. Future studies will examine what the long-term effects of the COVID-19 pandemic on intergenerational relationships are, and how intergenerational solidarity and conflicts might have been affected by the direct and indirect consequences of the pandemic.

In terms of policy implications, our study clearly demonstrates the importance of technologies that allow older people to maintain social contacts also at a distance. Other studies have found that non-physical social contacts have been crucial for reducing the negative mental health consequences of lockdown on older people (Arpino et al. 2020a), suggesting that the increase in nonphysical contacts that we document in this paper had in part compensated for the reduction in physical social contacts. The heterogeneity that we find in changes in nonphysical intergenerational contacts is probably in part related to technological divides in terms of age and socioeconomic status (see, e.g., Huxhold et al. 2020). Policies and interventions devoted at reducing existing digital divides may help older people to keep intergenerational (and other) contacts at a distance which is particularly relevant for older individuals who have to cope with situations of isolation and may result especially crucial in the future, in case similar lockdown measures will be required.

## Supplementary Information

Below is the link to the electronic supplementary material.

Supplementary file1 (DOCX 314 KB)
