# Supplementary material for: Physically distant but socially close? Changes in non-physical intergenerational contacts at the onset of the COVID-19 pandemic among older people in France, Italy and Spain
Source: Eur J Ageing. 2021 Apr 26;18(2):185–94. doi: 10.1007/s10433-021-00621-x (PMC8074698; doi:10.1007/s10433-021-00621-x)
Supplement: Supplementary file 1 — Supplementary file1 (DOCX 314 KB) [file 10433_2021_621_MOESM1_ESM.docx]

**Physically distant but socially close?**

**Changes in non-physical intergenerational contacts at the onset of the COVID-19 pandemic among older people in France, Italy and Spain**

Bruno Arpino^1^, Marta Pasqualini^2^, Valeria Bordone^3^

^1.^ Department of Statistics, Computer Science, Applications, University of Florence, Viale Morgagni, 59, 50134 Firenze, Italy. [bruno.arpino@unifi.it](mailto:bruno.arpino@unifi.it). ORCID: 0000-0002-8374-3066 (Corresponding author)

^2.^ Observatoire sociologique du changement (OSC), Sciences Po (Paris). [marta.pasqualini@sciencespo.fr](mailto:marta.pasqualini@sciencespo.fr). ORCID: 0000-0002-8379-375X

^3.^ Department of Sociology, University of Vienna, Rooseveltplatz 2, 1090 Vienna, Austria. [valeria.bordone@univie.ac.at](mailto:valeria.bordone@univie.ac.at). ORCID: 0000-0002-2987-3978

**Supplementary Materials**

**Table S.1. Cross-tabulation of the distribution of the sample by changes in physical and non-physical contacts**

|  | PC increased | PC stable | PC decreased |
| --- | --- | --- | --- |
| NPC decreased | 163 | 344 | 1295 |
| NPC stable | 23 | 381 | 54 |
| NPC increased | 253 | 1269 | 425 |

Note: N = 4,207. Source: Intergen-covid online survey. Data were collected between 14-24 April 2020

**Table S.2. Association Between Changes in Physical Intergenerational Contacts and Non-physical Intergenerational Contacts During the Lockdown (Model 2). Robustness Check**

| **VARIABLES** | **Model 2** | |
| --- | --- | --- |
|  | beta (SE) | |
| Decreased physical intergenerational contacts (all) | 2.062*** | (0.110) |
| Age | -0.140 | (0.108) |
| Age squared | 0.001 | (0.001) |
| Gender: Female (ref. Male) | 0.080 | (0.109) |
| Educational level: Medium (ref. High) | 0.305* | (0.180) |
| Educational level: Low (ref. High) | 0.224 | (0.218) |
| Country: Italy (ref. Spain) | -0.269* | (0.150) |
| Country: France (ref. Spain) | -0.205 | (0.169) |
| Income: Living comfortably on present income (ref. Coping on present income) | -0.130 | (0.153) |
| Income: Finding it difficult on present income (ref. Coping on present income) | -0.316** | (0.135) |
| Income: Finding it very difficult on present income (ref. Coping on present income) | -0.029 | (0.267) |
| Self-rated health: Poor (ref. Good) | -0.027 | (0.125) |
| Chronic diseases: Yes (ref. No) | 0.142 | (0.126) |
| Employment status: Yes (ref. No) | 0.063 | (0.130) |
| Childless & Grandchildless (ref. Children only) | -1.192*** | (0.142) |
| Children & Grandchildren (ref. Children only) | 0.218 | (0.139) |
| Parents alive | 0.701*** | (0.113) |
| Partner alive | 0.172 | (0.129) |
| Reduction in physical activity | 0.479*** | (0.132) |
| Worsened relation with partner | -0.246 | (0.226) |
| Worsened relation with other people | 0.180 | (0.188) |
| Suffered income loss | 0.0125 | (0.138) |
| Lost job | 0.160 | (0.294) |
| Difficulties with organizing work or study from home | -0.130 | (0.219) |
| Death of a relative or friend due to Coronavirus | -0.253 | (0.229) |
| A relative or friend was infected | 0.269* | (0.162) |
| Had more time to spend with family | -0.149 | (0.132) |
| Made new friends | -0.216 | (0.365) |
| Re-established a relationship with a relative or friend | 0.272* | (0.155) |
| My life was not affected a big deal | -0.072 | (0.194) |
| None of the above | -0.041 | (0.284) |
| Number of COVID-19 cases in the region of residence: Second tertile (ref. First tertile) | -0.199 | (0.136) |
| Number of COVID-19 cases in the region of residence: Third tertile (ref. First tertile) | -0.299* | (0.163) |

Note: N = 3,333. Post-stratification weights are used. Robust standard Error (SE) in brackets Source: Intergen-covid online survey. Data were collected between14-24 April 2020.

**Table S.3. Descriptive statistics for control variables (%)**

| **Variables** | **Categories** | **Total** |
| --- | --- | --- |
| **Age (Mean, SD)** |  | 64.65 (8.98) |
| **Gender** | *Women* | 52.06 |
| **Educational level** | *High* | 14.46 |
|  | *Medium-* | 69.87 |
|  | *Low* | 15.67 |
| **Employment status before COVID-19 pandemic** | *Employed* | 31.71 |
| **Income before COVID-19 pandemic** | *Living comfortably on present income* | 17.01 |
|  | *Coping on present income* | 52.29 |
|  | *Finding it difficult on present income* | 23.13 |
|  | *Finding it very difficult on present income* | 7.57 |
| **Self-rated health** | *Poor* | 47.01 |
| **Chronic condition** | *Any* | 42.74 |
| **Family Structure** | *Partner* | 66.52 |
|  | *Parents* | 29.81 |
|  | *Childless & Grandchildless* | 24.87 |
|  | *Children only* | 41.17 |
|  | *Children & Grandchildren* | 33.96 |
| **Experiences during COVID-19 pandemic** | *Reduction in physical activity* | 48.18 |
|  | *Worsened relation with partner* | 4.92 |
|  | *Worsened relation with other people* | 7.17 |
|  | *Suffered income loss* | 26.04 |
|  | *Lost job* | 3.35 |
|  | *Difficulties with organizing work or study from home* | 6.07 |
|  | *Death of a relative or friend due to Coronavirus* | 8.77 |
|  | *A relative or friend was infected* | 14.58 |
|  | *Had more time to spend with family* | 30.49 |
|  | *Made new friends* | 2.59 |
|  | *Re-established a relationship with a relative or friend* | 12.45 |
|  | *My life was not affected a big deal* | 23.18 |
|  | *None of the above* | 6.06 |
| **Number of COVID-19 cases in the region of residence** | |  |
|  | *First tertile* | 31.45 |
|  | *Second tertile* | 32.60 |
|  | *Third tertile* | 35.95 |

Note: N = 3,333. Post-stratification weights are used. SD: Standard Deviation

Source: Intergen-covid online survey. Data were collected between14-24 April 2020

**Figure S.1 Predicted probabilities of increased Non-physical Intergenerational Contacts during the COVID-19 lockdown by age (Model 1)**

**
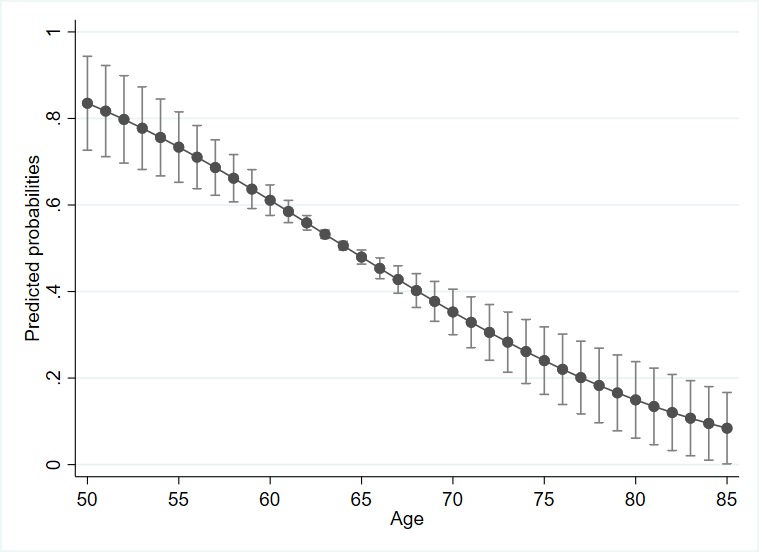
**

Note: N = 3,333. Post-stratification weights are used. Control variables included in the models. Source: Online survey implemented by the authors. Data were collected between14-24 April 2020.

**Figure S.2 Predicted probabilities of increased Non-physical Intergenerational Contacts during the COVID-19 lockdown by family structure (Model 1)**

**
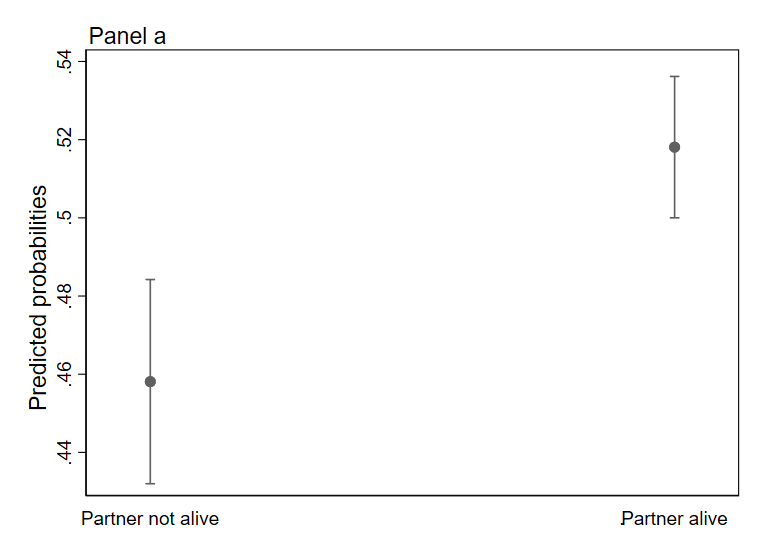
**

**
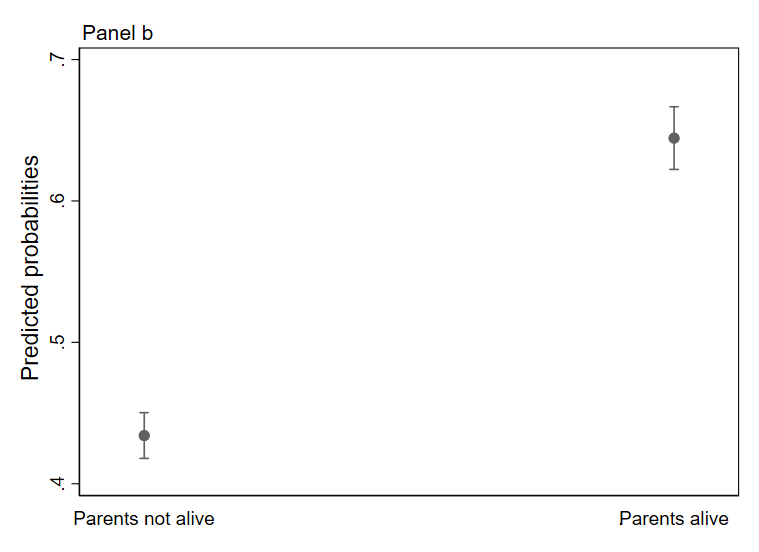

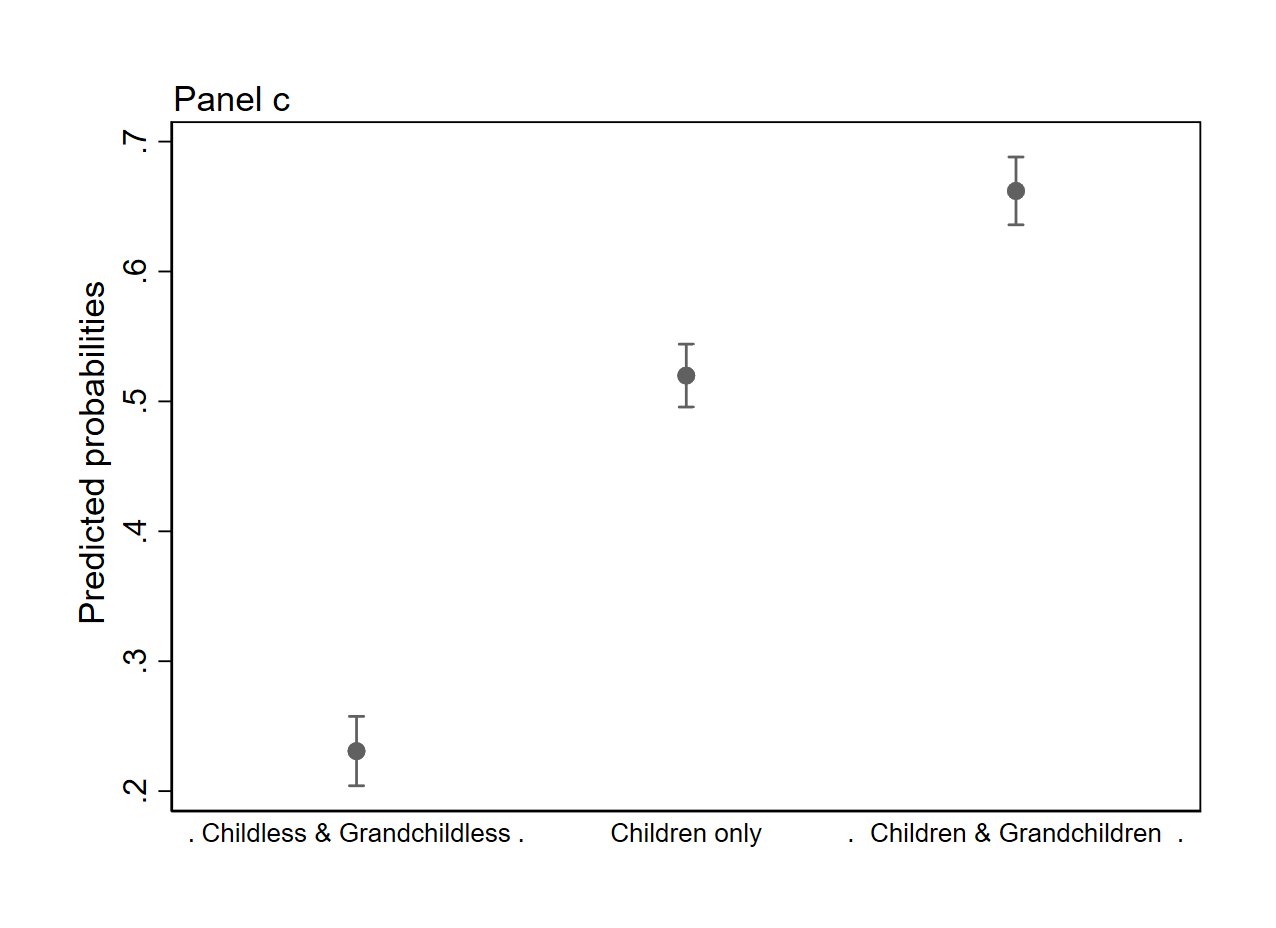
**

Note: N = 3,333. Post-stratification weights are used. Control variables included in the models. Source: Online survey implemented by the authors. Data were collected between14-24 April 2020.

**Figure S.3 Predicted probabilities of increased Non-physical Intergenerational Contacts during the COVID-19 lockdown by changes in physical intergenerational contacts (Model 2)**


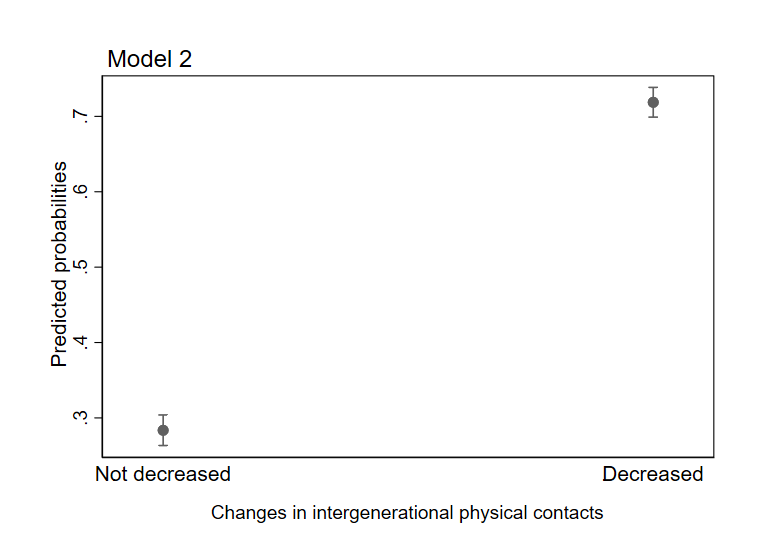


Note: N = 3,333. Post-stratification weights are used. Control variables included in the models. Source: Online survey implemented by the authors. Data were collected between14-24 April 2020.

**Figure S.4 Predicted probabilities of increased Non-physical Intergenerational Contacts during the COVID-19 lockdown by changes in physical intergenerational contacts (Model 2a, Model 2b and Model 2c)**

**
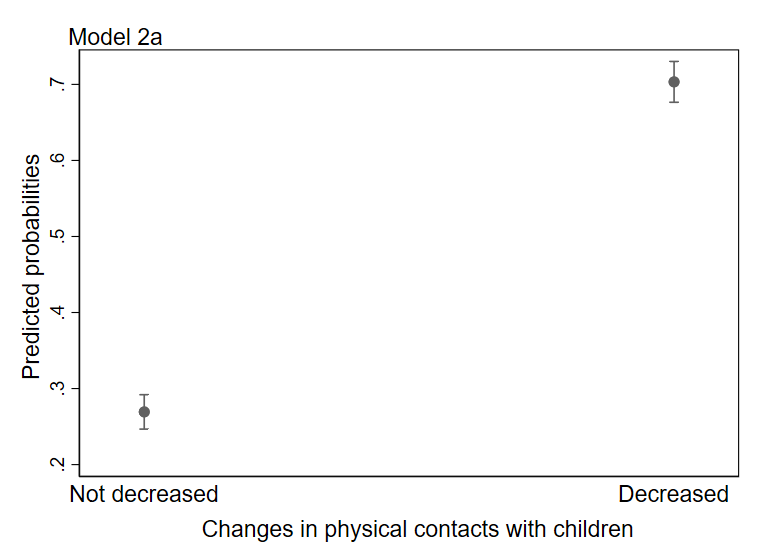

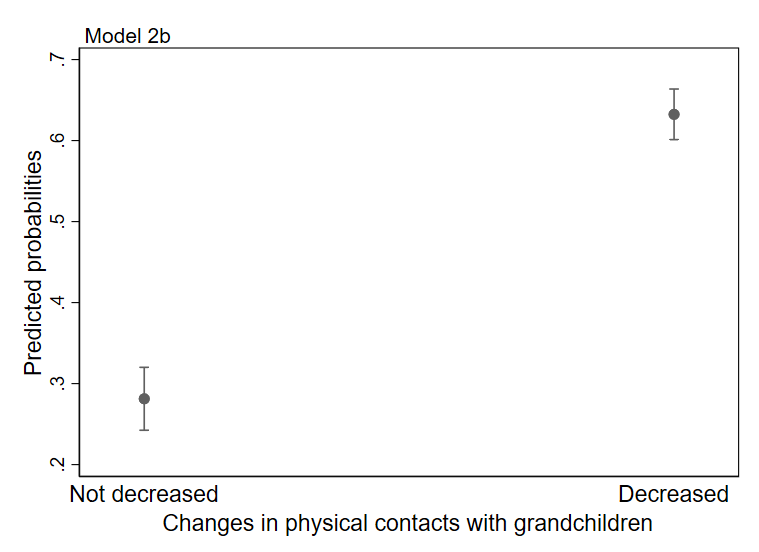
**

**
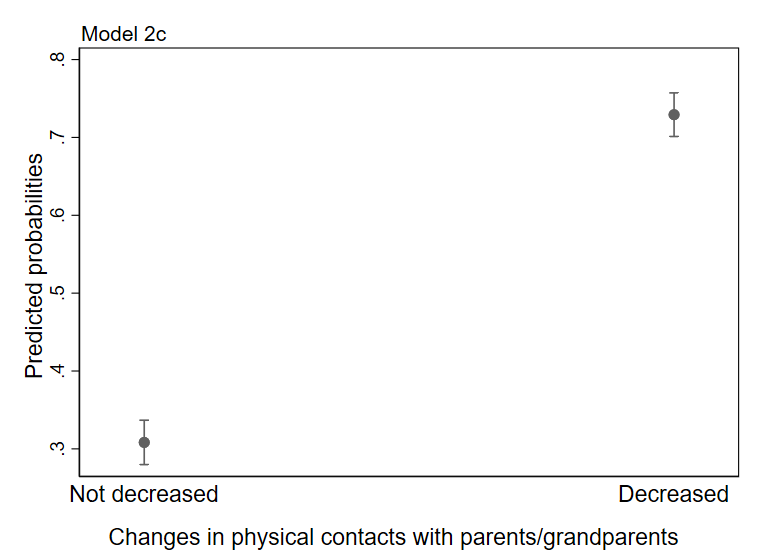
**

Note: Model 2a, N = 2,397; Model 2b, N = 1,105; Model 2c, N = 1,109. Post-stratification weights are used. Control variables included in the models.

Source: Online survey implemented by the authors. Data were collected between14-24 April 2020.

**Figure S.5 Predicted probabilities of increased use of digital devices during the COVID-19 lockdown by changes in physical intergenerational contacts (Model 3: video calls, Model 4: instant messages and Model 5: social networks)**

**
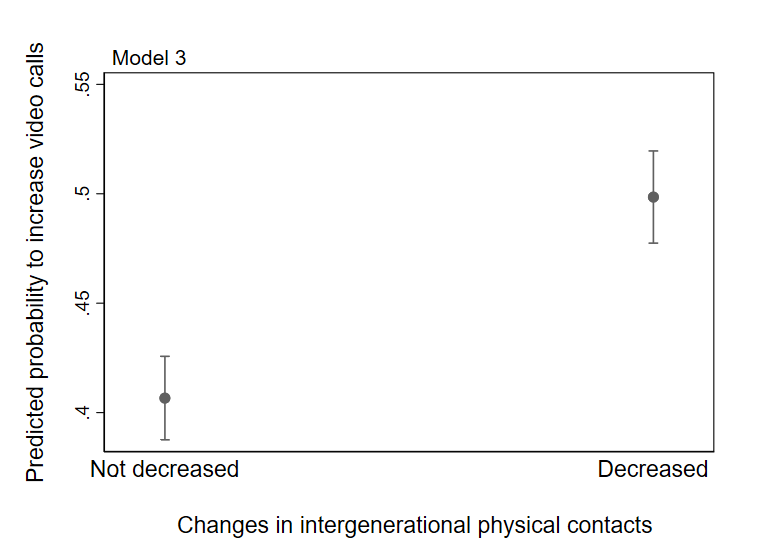
**

**
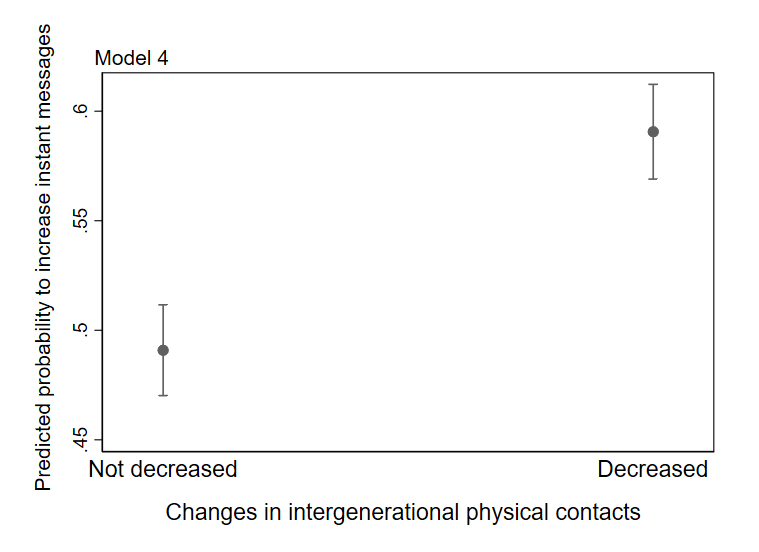
**

**
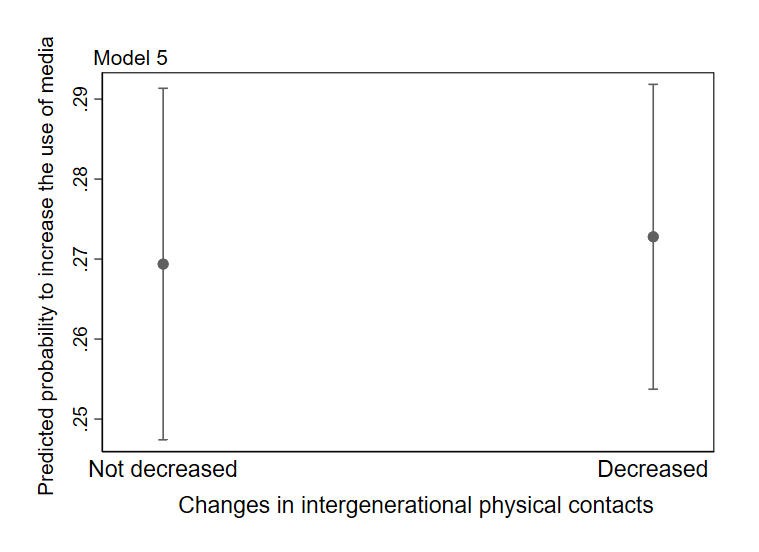
**

Note: N = 3,333. Post-stratification weights are used. Control variables included in the models.

Source: Online survey implemented by the authors. Data were collected between14-24 April 2020.
